# I4.0 Pilot Project on a Semiconductor Industry: Implementation and Lessons Learned

**DOI:** 10.3390/s20205752

**Published:** 2020-10-10

**Authors:** Gabriel do N. Silveira, Rafael F. Viana, Miromar J. Lima, Henrique C. Kuhn, Cesar David P. Crovato, Sandro B. Ferreira, Giovani Pesenti, Erick Storck, Rodrigo da Rosa Righi

**Affiliations:** 1 Technological Institute on Semiconductors, Unisinos University, São Leopoldo 93022-750, Brazil; gnascimentos@unisinos.br (G.d.N.S.); rafaelviana@edu.unisinos.br (R.F.V.); miromar@edu.unisinos.br (M.J.L.); cafrunikuhn@edu.unisinos.br (H.C.K.); ccrovato@unisinos.br (C.D.P.C.); sbinsfeld@unisinos.br (S.B.F.); 2 HT Micron Semiconductor S.A, São Leopoldo 93022-750, Brazil; giovani.pesenti@htmicron.com.br (G.P.); erick.storck@htmicron.com.br (E.S.)

**Keywords:** pilot project, Industry 4.0, IoT, flexible solutions, semiconductor factory, critical variables

## Abstract

Industry 4.0 considers the combinations of Internet of Things, computing and communication infrastructure, sensors, and artificial intelligence (AI) to provide predictive maintenance and process optimization. These benefits are very relevant to the semiconductor industry, where high reliability and low operating costs are critical for a business’ success. Analyzing the state-of-the-art of the projects that present the implementation of the fourth industrial revolution in semiconductor companies, we noticed mainly two branches of initiatives: (i) articles that explain the final achievements, not detailing how they were assembled and structured; (ii) articles that detail a part of the industry ecosystem, for example, approaching only the communication system or AI algorithms. In this context, this article proposes an I4.0 Pilot as a compilation of lessons learned during an end-to-end development of a reference design applied to a semiconductor packaging and test company. We explore the requirements of clean rooms and information related to sensors and data acquisition boards, in addition to performance details and configurations pertaining to visualization tools and warning notifications. The main contribution appears in presenting the advantages of adopting flexible decisions in the pilot to enable the best characteristics for a final expandable solution. Our final idea is to emphasize the importance of having a pilot project without significant expenses, presenting the reader with the acquired knowledge, and how they can benefit from it.

## 1. Introduction

In the semiconductor industry, technology and process reliability are crucial factors in ensuring product competitiveness and quality. In this context, Industry 4.0 (I4.0) appears as a combination of the Internet of Things (IoT) and Artificial Intelligence (AI), contemplating an ecosystem ranging from acquisition of data to the extraction of knowledge. Sensors, actuators, data acquisition boards, gateways, and back-end servers (cloud-based or on-premises solutions) work together to save raw data continuously in appropriate databases [1,2,3,4]. Thus, they allow the application of AI techniques to provide data prediction, pattern recognition, and event correlation in favor of predictive maintenance and insights that can be useful to obtain a competitive advantage. In this way, I4.0 can increase connectivity between different sectors of a company, showing relationships never seen before, also improving process analysis and complex decision making.

In the context of semiconductor companies, I4.0 generates a clear commercial advantage over competitors. From wafer cutting to chip testing, the entire manufacturing process can be monitored and improved with greater speed [5,6]. As the semiconductor business operates with high yield and high cost, consistent and reliable monitoring of critical variables becomes an essential requirement in the economic and productive scenario. Data monitoring and application of AI techniques allow us to analyze the functioning of the packaging process steps more closely, such as wafer backgriding, wafer dicing, die-attach, wire bonding, chip molding, and electrical test. Moreover, clean rooms, where these different production stages occur, demand full-time monitoring in relation to the number of particles and the humidity of the air. This all demonstrates the need for I4.0 applications in this industrial segment.

Several factors should be taken into account when implementing a Hardware and Software project for a semiconductor company. In our case, one of the main concerns is the flexibility of the project. In particular, the data acquisition system’s architecture must be selected based on the specification of the connectivity protocol. This is a significant project decision, as it defines implementation strategies. As each plant has its layout concerning machine distribution, access to resources, and infrastructure complexity, large-scale implementation of the I4.0 project takes time to collect valuable results [4]. Design adjustments in this scenario can also be costly in terms of human resources, time, and budget. To avoid these complexities, improving the time to market for high-quality products, pilot projects emerge as a useful strategy to experiment with I4.0 more quickly.

Our I4.0 state-of-the-art review found no initiatives that detail steps procedures for running an end-to-end project. Although there are several reference designs and architectures for the entire I4.0 framework, we have not observed a specific guideline covering a complete I4.0 deployment. Neither one presents flexible solutions, with options to choose from for the different situations encountered in the field at the implementation time. Our I4.0 Pilot Project (I4.0 Pilot) explains the decisions for each area, highlighting each way’s impact on the project’s future stages. Our strategy is to divide a project into smaller and faster implementation stages, allowing for more rapid assessment and correction before executing a large-scale solution. Although it applies to a semiconductor company, the ideas presented serve as a basis for entrepreneurs to model I4.0 projects, taking advantage of all the benefits mentioned above. The contributions of the article are twofold.

- We present I4.0 Pilot as an end-to-end methodology to develop an Industry 4.0 Pilot project, highlighting each area and its major decisions.
- From a technical viewpoint, we have provided an I4.0 Pilot in a Brazilian-Korean semiconductor industry. Here, we present the technologies adopted, a discussion about the lessons learned, which paths could be followed, and which ones should not.

After presenting the introduction in Section 1, Section 2 reveals the related works and current gaps in the joint combination of I4.0 Pilot projects and semiconductor companies. Section 3 describes the I4.0 Pilot, detailing each area of the proposal, while Section 4 discusses the results and lessons learned. Section 5 emphasizes the scientific contribution of the work and notes several challenges that we may face in the future.

## 2. Related Work

Some works have already been conducted in the Industry 4.0 IoT technologies field. In [7], the authors explored the steps to implement IoT approaches in a smart factory environment. They compared different methodologies and techniques, providing a reference architecture to the practical implementation of intelligent manufacturing. Moreover, the work studied the link between automating the factory and the production process, taking into account information system architecture and real-time analysis. Similarly, Yan et al. [8] implemented a framework for structuring and characterizing industrial big data and manufacturing process aspects. The research also investigated big data processing, involving predictive maintenance, energy-saving, and industrial facilities.

In [9], the author described an approach to transform a legacy factory into a smart one, using I4.0 principles to accomplish this. It was described as a multi-phase, multi-year implementation plan. Each year or phase introduces new technologies onto the factory, varying from ERP (Enterprise Resource Planning), MES (Manufactoring Execution system), etc. at the first phase, automation and seamless integration in the middle stages to achieving IoT and real-time analytics in the last step. The work described solutions, implementation approaches, and challenges faced in each phase. The results in [10,11,12] presented a cyber-physical system (CPS) architecture for I4.0 for manufactures, detailing five levels of technologies ranging from connected sensors to self-adaptive supervisory control. Ref. [10]’s main contribution is a middle layer that proposes an interconnected machinery layout, providing a self-comparison ability that allows quicker performance comparison between machines and behavior prediction. Prist et al. [11] presents a software architecture and cloud computing layer as an approach to enable cyber-physical manufacturing systems on legacy production line machinery. The proposed architecture acts as an intermediate layer between target devices to establish a flexible and independent network. Its scope ranges from physical sensors, network devices to cloud computing and data analysis. A pilot case is presented, showing the proposed architecture efficacy. Analyzing the initiatives mentioned earlier, we perceive that most of them explained aspects related to IoT communication in terms of optimizations in the communication protocol and middleware to implement data capturing and transmission easier [10,13]. Frameworks that explore modules and their interconnection are the main focus of [7,8]. They also discussed artificial intelligence and big data techniques to get insights over collected data and provide predictive maintenance.

In [9,14,15], we have initiatives that presented a transformation of a non-I4.0 industry in an I4.0-based one. Theses works propose the I4.0 implementation for semiconductor factories in phases without emphasizing sensors or big data techniques. These works also refer to a complete translation from one kind of industry to another, not testing and evaluating different aspects of technologies and decisions in the short-term as a pilot project does. In [16], the authors built a complete prototype Industrial Internet of Things (IIOT) system for use at surface mount technology (SMT) assembly line. An IoT end-devices (IEDs) containing vibration, proximity, and temperature sensors gather data periodically by reading the sensors attached to them and relay them to the IoT Gateway over a suitable protocol (I2c, BLE, MODBUS, etc). The end devices talks to the IoT Gateway (IGW) using respective drivers, which then forwards the data to an MQTT Broker (using open-source Mosquitto) and stores them in a message queue. This data is then sent to a Middleware server (database) over HTTP REST API for data analysis.

In terms of communication, the study in [17] exposed seamless resources sharing architecture involving IoT methods, such as cloud data transference and wireless network approaches, with the intent to improve performance. The authors also simulated scenarios with Matlab software to observe three communication technologies (5G, 4G, and IEEE 802.11), considering different QoS requirements to monitor the proposed solution’s behavior. The search also englobed VoIP and video application to simulate an environment where jitter and delay metrics are essential. Overall, best-effort services are studied to evaluate if the proposed architecture deals with QoS necessities. Keeping the scope of communication protocols, in [13], the authors studied a subset of IEEE 802.15.4 configurations with the intent to improve performance, reliability, throughput, and power management of a group of network topologies. The researches explored IEEE 802.15.4 PHY and MAC specifications and the Zigbee routing layer. On the other hand, in [18] describes the implementation of the sensor node, focusing on the multi-protocol with LoRaWAN and Wi-Fi connectivity options. In [19], the authors address the leading potential technologies currently for IWSAN solutions, as well as their advantages and disadvantages.

In this way, we perceive a gap in the literature toward providing an end-to-end methodology to implement I4.0 for scratch following a pilot project’s principles. In other words, the state-of-the-art does not present articles that contemplate methods that present the phases of small-scale I4.0 implementations used to prove the viability of a project idea.

## 3. Reference Design on Developing A Pilot Project for A Semiconductor Company

This section presents our proposal, which can be seen as a reference design on developing a pilot project called Intelligent Factory for our customer, HT Micron Semiconductors S.A. We modeled this pilot with flexibility in mind so that adaptations for easy installability, reliability, and scalability can be made possible without compromising the project. The next subsection shows how this pilot was structured, starting with the customer’s definitions hand out.

### 3.1. Requirements and Functioning

The project aims at optimizing the operational cost of microelectronics factories by using the advent of the Internet of Things (IoT). It is essential to bear in mind that maintaining a clean room in operation is very expensive, and any unscheduled stops can cause high losses. The project has three slogans that guide it: the factory can never stop; reduce downtime and operating costs; be a product in the future. Therefore, we proposed to develop a monitoring system for critical variables in production equipment, electromechanical facilities, and power quality, which would be low-cost, low power, flexible, scalable, and use on-premise resources. In particular, this last topic is essential for security concerns, so remote access is not allowed in this context.

The development comprises three significant areas: hardware, firmware, and software for data monitoring; integrating board, wireless communication, computational resources, database, data analysis; and visualization. The main objective is to detect variations that may indicate operation or maintenance problems, allowing the maintenance team to act proactively, correlate sensor data, and get insights and patterns from raw data. We developed a flexible-driven pilot to test an end-to-end solution before replicating it to the entire clean room. This idea is pertinent not only for cost issues but also for training with different sensor load scenarios, radiofrequency protocols, and backend computing resources.

**Figure 1:**
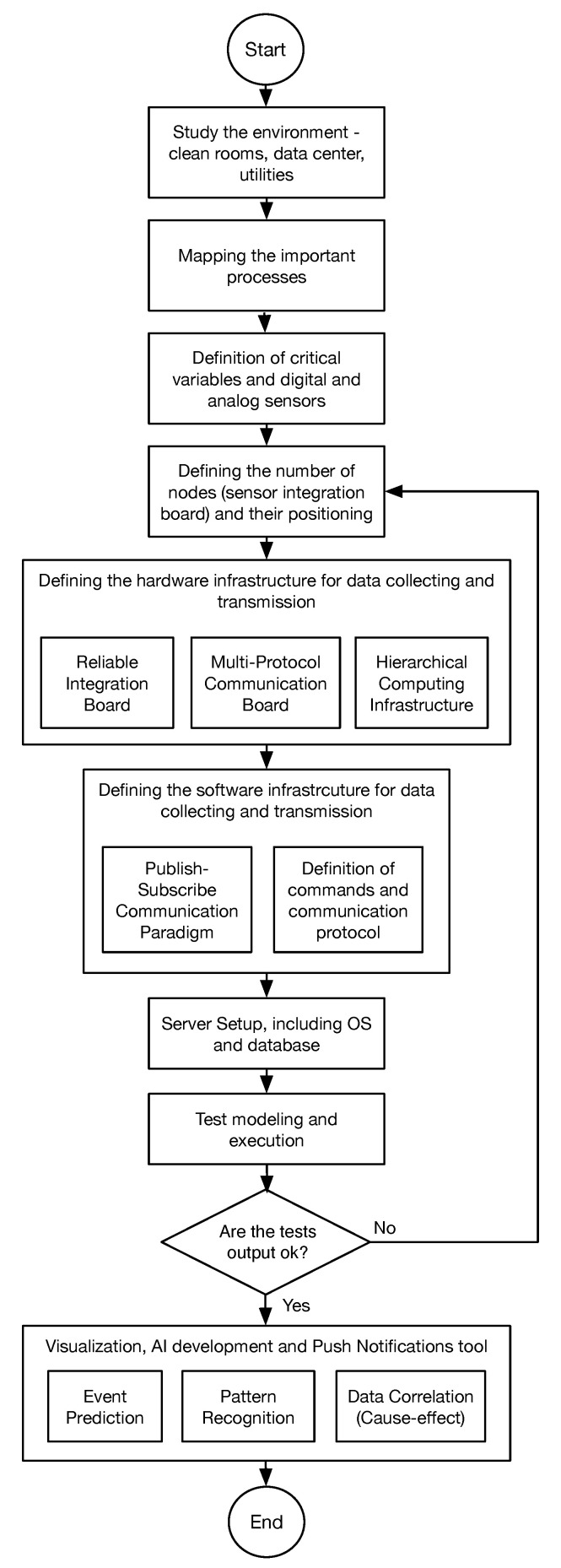
Pilot 4.0 reference design flowchart.

Figure 1 depicts an end-to-end organization of the proposed model to execute the pilot project. First, we need to study the environment of the industry. In particular, it is important to understand details about the functioning and the processes related to semiconductors. We must also observe the machines and engines in the clean rooms, data centers, and utilities room. After that, we define critical variables and digital and analog sensors to capture these variables. Here, together with the company, we need to observe those variables that directly impact the quality, the cost, and the risks of developing semiconductor products. Understanding the variables and the environment, the idea is to create the points where the measures will be collected. Furthermore, we need to create a hardware infrastructure involving an integration board (which captures data from sensors), a multi-platform communication board, and a hierarchical computing infrastructure. A hierarchical infrastructure is useful to have better system scalability. So, we modeled the type of sensors, integration boards, hubs or concentrators, and a central server. Users access this server to collect that, also receiving push notification from it.

The server runs a visualization tool and artificial intelligence algorithms, enabling the user to analyze historical data and observe the current functioning of the monitored clean rooms. As explained in Section 2, the existing literature in the semiconductor industry’s joint-area and Industry 4.0 does not present an end-to-end path to implementing an I4.0 project. Furthermore, we observe the lack of discussions about technologies and flexible adoptions and their impact on developing pilot projects. In this way, the next subsections present some detail about how we modeled our reference design for the pilot project, highlighting the impact of adopting flexible decisions, which can reduce risks of deploying the industrial plant project.

### 3.2. Critical Variables

The critical variables are identified and classified in joint-effort of the customer and the development team. In the example below, three classes of variables are defined: process, power quality, and machine quality variables. Process variables are those that are used or interfere with the quality of the different manufacturing processes of the semiconductor factory, such as: wafer backgrinding, dicing, die-attach, wire bonding, molding, solder ball attach, laser marking, singulation, burn-in, testing, and automatic visual inspection. The process variables can be divided into six groups: Process Cold Water (PCW), Clean Dry Air (CDA), Deionized Water (DI), Environment (ENV), Nitrogen (N2), and Vaccum (VAC). In particular, the nitrogen application can comprise both gaseous and liquid state. Table 1 presents a possible selection of target variables, with the respective sensor specifications that can be monitored. The power quality variables comprise the monitoring of voltage, current, power, and disturbances in factory electrical installations. Machine quality variables are associated with motors and dicing, monitoring their temperature and vibration. While these variables are also part of the project and affect development choices, this article focuses only on the process variables.

All groups, except the Environment (ENV), have their sensor elements mechanically connected to pipes. For these variables, we selected 4–20 mA transmitters because they are adaptive to many industrial devices. These transmitters are consolidated as an industry standard and have high immunity to background noise, low measurement uncertainty, easy detection of failures, and practical installation. The Environment (ENV) application is essential for the company, as it interferes with its quality. It comprises the monitoring of temperature, humidity, and particles in its clean rooms. Due to the application’s characteristics, we chose sensors that work with SPI and I2C protocols due to its interoperability with most microcontrollers. With this, it is possible to use a high data rate, buffering memory, and processing and more efficient transmission.

### 3.3. Integration Board

As presented in Section 3.2, the I4.0 Pilot consists of several sensors with different communication protocols to be monitored and integrated into an RF network. Although the sensors are spread throughout the factory, we grouped them, reducing the total number of nodes that had to be implemented. With that, two demands emerged: creating a board to integrate the different sensors and necessary functionalities and the choice of wireless MCU.

**Figure 2:**
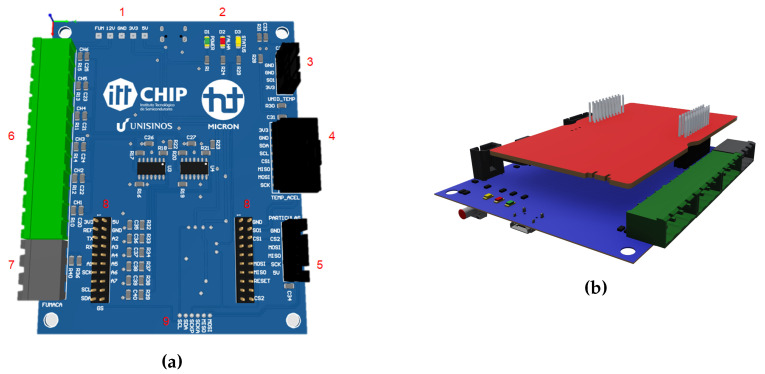
(**a**) Integration board that is able to receive data from up to 16 sensors; (**b**) IoT node composed of an integration board and a communication platform.

We chose the Texas Instruments Launchpad CC2650 (LP) [20] because it supports the connection of these various sensors. Furthermore, it enables the choice of multi-protocol for data communications, enabling the selection of low and medium data rates depending on the requirements, besides being low power. Opting for a development board helped to speed up the implementation. However, it was necessary to place signal conditioning circuits, suitable connectors, and a power supply to integrate all sensors and the LP. To achieve this, we developed the Integration Board illustrated in Figure 2a, acting as an extension of the LP’s functionalities, bringing robustness and security to the acquired data. The connection between the Integration Board, the LP, and the sensors are called the IoT Node, illustrated in Figure 2b. The IoT Node can support up to six analog transmitters and three digital sensors.

The Integration Board (Figure 2a) has the following features: (1) Test points for measuring power signals from the power supply’s and LP’s circuits. (2) LEDs for visualization, where: green indicates the presence of power; red indicates power failure, and yellow indicates data transmission status. (3) Connector for temperature and humidity sensors. (4) Connector for vibration and infrared temperature sensors. (5) Connector for particle sensor. (6) Connection of six 4–20 mA analog sensors. (7) Backup power supply. (8) Headers for LP connection. (9) Test points for measuring I2C and SPI digital signals.

### 3.4. Communication Platform and Protocol

**Figure 3:**
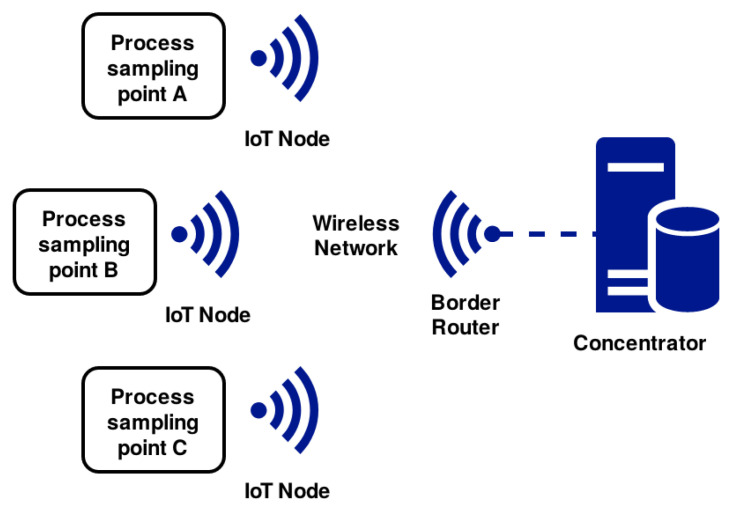
Network Architecture I4.0 Pilot project.

The communication architecture of the I4.0 Pilot is illustrated in Figure 3. The IoT Nodes refer to the device responsible for physical sampling data from the sensors and send it via a wireless connection, as described in Section 3.3. The Border Router plays the role of receiving data from the IoT Node and transmit it via the UART protocol to the concentrator. The wireless MCU chosen is used in both the IoT Node and the Border Router. The LP supports both Bluetooth Low Energy as IEEE 802.15.4 @ 2.4 GHz radio solutions, enabling the usage of the 6LowPAN wireless protocol.

The network operates in a star topology. Our idea is to operate with a reduced number of connected devices in the system initially to evaluate the 6LowPAN practical operational limits. All nodes were placed near one another, to increase the network signal’s strength and network quality. This approach allows for quick deployment of the proposed IoT solution, given that it requires minor changes in the manufacturing process due to the small size of the implemented devices. It also allows a practical test on the target environment, allowing acknowledging problems in the initial phases of the project and providing better guidance on future decisions of the final solution.

To implement the network stack and the user application, we selected the Contiki real-time operating system (RTOS), which enables the flexible design of embedded applications and configuration schemes, ranging from a simple node that sends a single sensor data periodically as well to a fully-customizable device that supports multiple sensor setups and sampling interval configuration. As the device was intended to work in an environment containing a significant amount of metal surfaces and machinery, which are obstacles to radio frequency propagation, the network configuration approach chosen in this project allowed a simple and reliable connection between nodes while keeping the bandwidth available to all devices. The target application developed under the Contiki OS was conceived regarding low network usage while maintaining the sampling communication requisite.

For future scalability, the communication protocol between IoT nodes and server applications was a critical project specification. It needs to be flexible and reliable enough to allow multiple nodes to dynamically join and exit the network. It is also necessary to fully support the high throughput demands on the server node, and it needs to satisfy the embedded device intrinsic processing power limitation. The idea of using an MQTT-SN client on the IoT Node side is pertinent to meet the limited payload wall from IEEE 802.15.4. The broker and clients on the server are seamlessly connected to the IoT Node using a custom communication protocol. The target network needs to meet sensor data bandwidth usage and networking control, server application configuration, and status payloads to monitor the systems’ status.

### 3.5. Computing Infrastructure

**Figure 4:**
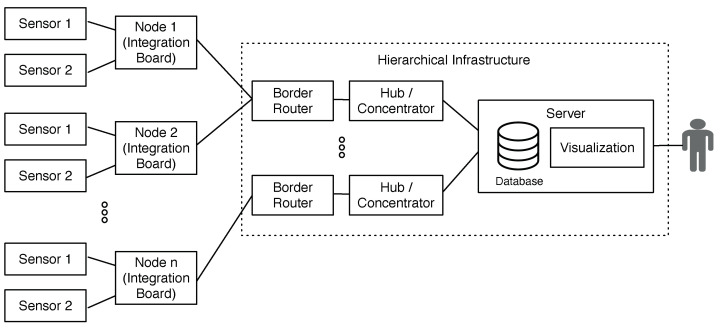
Pilot 4.0 network architecture, comprising an scalable and hierarchical infrastrcuture.

Figure 4 illustrates the computing infrastructure used for the I4.0 Pilot. As described in Section 3.4, the Border Router sends the data to the concentrator via USB. The concentrator is a minicomputer that transforms the incoming data in the engineering unit from sensors and turns it to its variable unit (e.g., bar) to store it in the database. From this point on, data could already be present to the user through dashboard resources. During implementation at the factory, some operational metrics about the RF network were observed. Among them, packet transmission and reception rates, and the maximum supported distance between the node and the concentrator. These were minimum requirements to ensure the robustness of the system for the next steps.

The minicomputer choice was a Dell OptiPlex with an Intel I7 processor, 8 GB of RAM, and 1 TB HD. We implemented an open-source relational database in the concentrator, PostgreSQL, which proved to be performative, robust, and reliable. In the backend application, we also implemented the MQTT client for event management and the application for converting the data into its final unit. In the I4.0 Pilot, the same device (concentrator) played the application server and database server’s role. In the future, a robust machine will be dedicated to the server role; thus, the concentrator will remain exclusively as the interface between the IoT Node and the server.

### 3.6. Programming Language and Communication Libraries

In the pilot phase, the user would directly register the IoT nodes, sensors, and configurations in the database, for practicality. This simplification process is reflected in the implementation speed, being careful not to compromise the quality and reliability.

The initial premise was that the programming language (PL) defined for the backend should also be used to implement the interface between the application and the application server, considerably reducing libraries and frameworks’ dependency management. Therefore, the PL should provide subsidies for both back-end programming, interacting with the computer’s network and operating system resources, and frontend, such as offering assistance to user interface development. Some criteria should be met, such as flexibility, interoperability, compatibility, and portability.

- Flexibility: The PL should allow the development of functionalities such as interacting with events in the MQTT network, enabling processing for measurement conversions and storage in a database.
- Interoperability: The PL should have a connection library for the database. It should also maintain compatibility with the central operating systems and have resources for implementing web interfaces, such as HTML, CSS, and JavaScript. It should allow the user to interact with the system through online forms.
- Compatibility: The PL should maintain compatibility with different development IDEs. It should also have resources to implement an MQTT client and a producer and consumer (publication and subscription) of the MQTT network events. In terms of compatibility, another feature should be implementing a layer between the application and the database, the so-called Object Relational Mapping (ORM).
- Portability: The PL should be cross-platform, allowing portability to other environments, if necessary. As it is a solution for the web platform, it must be portable for several devices, from pc, laptops, tablets, and smartphones—all of this in an agile way.

In addition to the requirements listed above, the PL should also be Open source and adopted by other companies within the IoT context. Moreover, preferably, the development team should be familiar with it. Ultimately, the PL adopted for backend and frontend was the Python programming language because it meets all the listed requirements. Its benefits are robust code, libraries [21], and its use in several application areas. Additionally to Python, we selected the Web2py framework, as it contains features that contribute to the productivity and quality of the final application.

### 3.7. Database

For the project, the database (DB) represents one of the primary resources. It plays an essential role in data storage and the interface for queries and other data processing applications. This includes predictions, artificial intelligence algorithms (AI), and techniques to identify patterns and correlations to extract new knowledge from these data. In the process of defining the database, the issues to be taken into account were separated into two groups: (i) the main, consisting of six requirements, with a functional perspective, that is, from an internal point of view and; (ii) the secondaries, composed of four other requirements, no less critical, but with an external perspective.

The primary requirements are: (1) Inseparability since the database should be integrated with other databases. (2) Compatibility with leading operating systems, such as Linux, Windows, and Mac OS. (3) Performance-oriented by being responsive to multiple continuous queries since this situation will define the real IoT situation. (4) Maintainability, which can be observed in terms of repairs, evolution, scalability, and reliability. (5) Usability, which is kept in two aspects: ease of learning and ease of use. (6) Security, which is evaluated in terms of supporting authentication, authorization, and cryptography.

The secondary requirements include four aspects. (1) Financial viability, the DB should be Open Source to minimize project costs. (2) Availability of technical support. Here, we considered necessary to have technological know-how available to support implementation. (3) Recent updates. The management tool should have updated versions released in the last 12 months. (4) Usability by large companies, which functions as a good indicator that the tool is reliable. Based on these requirements, the database administration tool was chosen: PostgreSQL. Several tests were also applied for performance analysis, evaluating them both in Windows and Linux environments. In conclusion, the performance in the Linux environment was significantly higher, since it presents itself as a superior tool for running as a server.

### 3.8. Deployment Issues and Test Modeling

Earlier subsections have presented critical variables, sensors, integration boards, wireless protocols, and our hierarchical computing organization with hubs/concentrators. Communication from sensors to the integration boards occurs through a wired connection. Each integration board transmits its data to a hub using radio frequency communication. This subsection presents our deployment modeling when considering the number of sensors per integration board, in addition to the acceptable number of this last kind of device that could be connected to each hub. The term acceptable here means the median situation where we have zero packet loss. In the 4.0 Pilot project, we are using IEEE 802.15.4, which is a technical standard that defines the operation of low-rate wireless personal area networks. It specifies both the physical layer and media access control (MAC) for 6LowPAN communications. This last establishes a binding for the IPv6 version of the Internet Protocol (IP) over WPANs.

**Figure 5:**
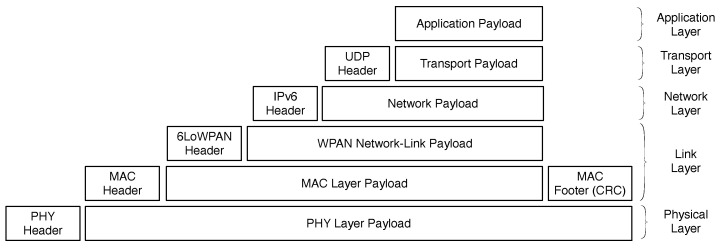
Communication stack from a particular integration board and a hub in the proposed architecture.

Figure 5 illustrates the communication stack from a particular integration board and a hub in the proposed architecture. To analyze the communication latency from one side the application layer from one point to the application layer on the other end, we understand that we have the sum of the following times: (i) overhead related to communication protocol processing; (ii) overhead to access the network, i.e., to insert a packet in the physical layer; (iii) transmission time in a wireless fashion. Considering (i), our protocol has a cost (which has the command type, a particular value measured by a specific sensor, and a timestamp) and the MQTT protocol payload (with ACK enabled). Respectively, we have 28 and 7 bytes for these aforesaid data. Furthermore, UDP adds 7 bytes, while IPV6/6LowPAN inserts the other 23 bytes. The MAC layer, which supports a maximum frame size of 127 bytes, adds 25 bytes in the communication, while the PHY layer inserts 6 bytes. In total, we have 97 bytes that are generated for each sensor per integration board. Our modeling is considering the same amount of bytes for the sensors, independently of their class.

Considering (i), we have the processing time of each layer on computing a particular header. For example, in the MAC layer, we have multiplexing management, frame validation, and acknowledged frame delivery. Furthermore, we have the Contiki operating system delay on transmitting data backward and forward between the layers. Regarding (ii), in the MAC layer, we have the carrier-sense multiple access with collision avoidance (CSMA/CA) algorithms to enable transmission of frames through the physical channel. In other words, it is not enough to get a ready frame to be sent, since we also have additional negotiations to access the physical layer and manage eventual problems on data sending. In (iii), the theoretical bandwidth limit of 250 Kbps (IEEE 802.15.4 at 2.4 GHz) must be respected. Considering these three sources of overhead on data communication and handling a fixed and equal-size number of sensors ($n_{s}$) per integration board, our idea is to maximize the number of integration boards ($n_{i}b$) that could be connected to each hub. To accomplish this, we have modeled Equation (1).

(1) $$n_{ib}=Max\;n_{ib}\;|\;\left(\frac{(n_{s}*total\_overhead*n_{ib})+Keep\_alive}{time}\right)\leq Bandwidth\;where\;packet\_loss=0$$

In Equation (1), total_overhead is equal to 194 bytes (97 bytes encapsulated in a packet of 125 bytes and an QoS-enabled ACK with a packet of 69 bytes). In addition, we have a keep alive message that is generated by the MQTT library, so causing an addition of 70 bytes for send this message and other 64 bytes to receive the ACK of this keep alive. Furthermore, time means the interval to send this data through the network. This equation only handles the transmission time, not addressing the delay on the processing layers and operating system. Thus, for example, adopting eight sensors and time equal to 1 s, we have a capacity close to 16 integration boards that could be allocated for a single hub in a 250 Kbps network. The larger the time interval, the worse the quality of the data monitoring and its usage on the artificial intelligence algorithm afterward (fault detection, for example). Our idea is to use the shortest time as possible, maintaining the zero packet loss constraint. Furthermore, Bandwidth in Equation (1) refers to the nominal limit of the IEEE 802.15.4 technology. In this way, In Section 4.1, we detail our tests to find $n_{ib}$. Although reaching a pertinent value for this variable, so we have zero packet loss, real tests revealed that sometimes packet loss could occur.

It is important to set n_ib and time at testing time. We can reduce n_ib or enlarge time, but these parameters are not easy to reconfigure at runtime because the first addresses physical devices while the second impacts the quality of the artificial intelligence algorithms. Thus, we modeled a buffer of 140 bytes (equal to 5 × 28, where 28 bytes is the amount of data for each transmission at the application layer, and 5 is the number of packets that we can buffer) at each integration board in such as way its content is only removed after we received the ACK on packet transmission. This ensures that we do not lose any data, ensuring the AI algorithms’ correct functioning afterward. Besides, each collected data has its own timestamp, which is really used in business applications instead of when the data communications happened.

### 3.9. Visualization Tool

**Figure 6:**
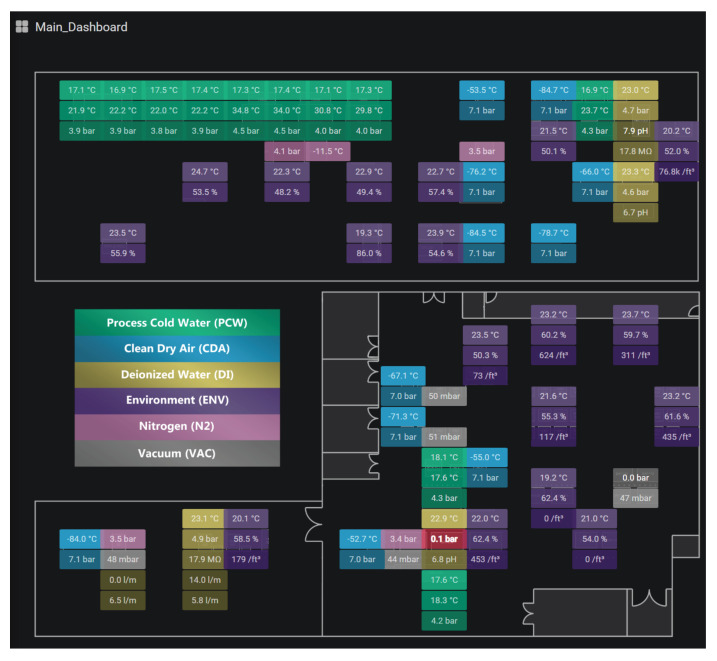
Using the visualization tool to map and analyze the state of the sensors in the industrial plant.

**Figure 7:**
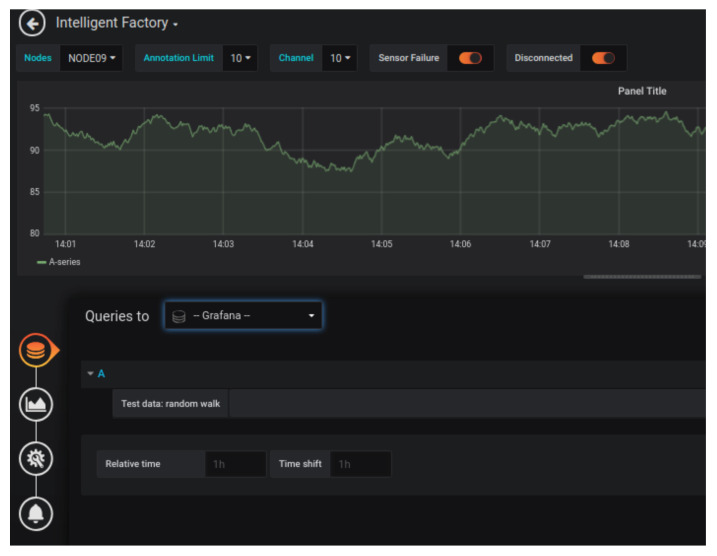
Example of observing historical data of a particular sensor. The graphical interface allows system administrators to configure different threshold-based warning levels, enabling push notifications that trigger actions to the support team.

As an essential requirement, the tool used to visualize the data should allow real-time monitoring and visualization of the event history. As the main criteria, the tool should allow the addition of plugins, allowing different visualization features. Moreover, the tool should be compatible with different databases and operating systems. The chosen solution was Grafana, a multi-platform, open-source web application for data analysis and interactive visualization [22]. In addition to meeting the mentioned requirements, large companies, such as Intel, Samsung, and Apple, also adopt it. The implementation involved making each sensor’s values available in the physical position in which the node is in the factory. Figure 6 shows a possible implementation of the visualization tool. Additionally, to better illustrate the functionality, Figure 7 presents the event history of a sensor. Visual alerts were also created in Grafana. In the same way, textual alerts were enabled via the Telegram application. The alerts can originate due to the measured value being outside the operational limits or data loss. This loss can occur either by disconnecting the node from the network or by disconnecting its power.

## 4. Results

This section first presents a discussion about performance and deployments tests and the learned lessons. First, we bring some performance details about the communication interval between integration boards and hubs, in addition to measures related to network latency and packet loss. Second, we give the impact of adopting flexible solutions and their impact on solving detected problems.

### 4.1. Deployment Details and Execution Performance

This subsection presents the initial testing when executing the Pilot project in the HT Micron Semiconductor company. The tests consisted of deploying a 6LowPAN network to connect the integration boards and the hubs. From the hubs to the central server, we have wired communication through a Gigabit Ethernet link. To analyze the impact of end-to-end communication (from data collecting on sensors up to saving data in the server database), we modeled ICMP packets of 65 bytes to generate a scenario close to the worst case of a MQTT packet size.

The tests considered eight integration boards (nodes) where each one receives the connection of two sensors (dew point and temperature). Both the integration boards and the hubs run MQTT software. Each integration board collects data from the sensors and transmits them to the hubs in an interval of 1 s. The percentage of packet loss was equal to 35%, and the mean network latency between the integration board and the hub is equal to 574 ms. The IEEE 802.15.4 protocol is not a high-speed network protocol since it focuses on low power devices. In this way, we increase the interval to send data from 1 s to 5 s. With 5 s, we do not have any packet loss so obtaining a communication latency of 135 ms. Here, the latency is better because network is not overloaded and we have a shorter delay on ACK handling. With this parameter, we can implement a larger number of integration boards without causing any collision and packet loss.

Considering Equation (1), the evaluation revealed that the best number of integration boards that can be connected in a single hub is equal to 8. This number allows us to have a pertinent communication latency and a reliable data collecting environment, respecting the requirement of zero packet loss. We also observed the added software layers do not affect the network performance. The use of CPU time in all the process (integration board, hub, and server) present a mean of 5%. Finally, we have tuned the communication parameters to have a pertinent number of data that allow the support team to understand event predictions, correlations, and fault models. Today, the project runs in the HT Micron Semiconductor industry, being valuable to control all the processes related to clean rooms. In particular, we configured the visualization tool to present different colors for each sensor and integration board, depending on the warning management, allowing the support team to solve the problems immediately.

### 4.2. Discussion and Learned Lessons

The development of a pilot is an excellent strategy both from a project perspective as a financial one. Through this strategy, it is possible to test elements that will remain in the solution, and others removed. At this stage, the client company’s stakeholders’ participation is important, making the solution unique. For this project, several lessons were learned, such as:

- Hardware: Plan with your team all the items that need to be documented. Simulate each step of the hardware and, finally, its integration. Review the components used in detail and, if possible, prototype the critical parts and test them. In this project, energy consumption and the correct and efficient handling of analog and digital signals were critical. The experience of the developers is also paramount in predicting some of the typical problems of these applications. The choice of sensors should be well planned, buy good sensors, and be tested in a controlled environment whenever possible. It is common to associate the failures to the hardware under development. Furthermore, try to buy from suppliers close to your customer for any future maintenance needs. It is recommended that factors such as financial resources and available manufacturing time are considered. It is interesting to first assemble some prototypes, between three and four parts, and then test them in your development environment, having the opportunity to correct layout and design errors. Finally, manufacture the quantity that will be installed.
- Firmware: The firmware contains basic configurations about the network. The deployment of smaller networks at the beginning of the project helps identify early connection issues that may appear due to the discrepancy between the development environment in which the network may be initially tested and the actual factory physical space. We have found network performance issues on the existing factory due to the machinery and physical availability of device allocation, which directly impacted the network. This allowed a quick definition of the target network size, in the number of nodes, to reach the required sampling interval on the final project. An initial pilot application is useful for testing the network stack and evaluating node-server communication, making necessary changes to the application protocol, and ensuring a functional data flow in the system. Besides that, the pilot application allowed us to reach insights about the device’s hardware limitation, which may directly impact the final application’s complexity and performance and the interaction between the target application and the network specifications. Lastly, the usage of established devices and operational systems is recommended due to the extensive community support and use-case examples, which may reduce the overall development time and reach a more remarkable result.
- Software: In the software development viewpoint, the requirements collection stage should not be neglected, as it reduces time spent re-writing code due to lack of communication. We also observed that before developing a solution using a particular platform, even with several positive technical factors for choosing it, it is essential to look for non-technical reasons. For example, some customer preferences can be subjective and need to be respected and considered. After completing the software implementation, the primary lesson is related to the assurance that the defined infrastructure and technologies such as programming language and DBMS were adequate. We also observed that the implementations met expectations. It is also worth mentioning that some problems occurred in the database server during the system validation period. In particular, we had problems related to the virtual machine used, resulting in data loss due to VM corruption. This experience provided maturity in terms of backup routines and data replication. Initially, we observed excessive consumption of VM resources, which led us to perform tests with Windows and Linux operating systems. After extensive testing and configuration adjustments, we found that Linux proved to be more appropriate in terms of performance and scalability.

A compilation of lessons learned and adaptations can be seen in Table 2. Integrating all hardware, firmware, and software areas were successful thanks to both parties’ efforts: the company and the development team. We currently have more than 130 sensors and 40 nodes installed at the factory, monitoring various variables, running prediction algorithms, statistical process control, compression, and software sensors. In the future, we plan on integrating this into a single solution, such as a System in a Package (SiP). The parties’ collaboration strategy was vital, mainly due to the difference between development and implementation environments. The customer’s role went beyond defining requirements, followed the installation, and identified the project’s errors very carefully.

## 5. Conclusions

This article presented a flexible pilot project methodology as well as a deployment example in the context of a semiconductor company. We offer the pilot’s steps, highlighting our adoptions and their impact in terms of flexibility in the pilot and the project that will be implemented later. Flexibility is essential since we have the following situations in an IoT project: (i) the in-house tests are different of those executed in the company because the environment and obstacles for radiofrequency propagations are different; (ii) scalability is another challenge because in the company we will have the pilot running 24 × 7, generating data that must be reliable to feed machine learning algorithms which will control the monitored variables.

In our understanding, although widely commented, we observe that it is common among entrepreneurs the difficulty of expressing in detail how to transform ideas into a real I4.0 project. This way, besides exploring flexibility, we present a technical overview of how to assemble an IoT project for a semiconductor company. We have confidence that the proposed pilot is not restricted to this kind of company. It is easily adaptable to other scenarios, thus extending our contributions. In terms of future work, we plan to present details about the development of the project in the company and new insights about energy monitoring in the industrial plant. Furthermore, we plan to explore machine learning algorithms and the digital twin concept to automatic perform remediation actions when observing a particular problem in a machine or sensor.

## Figures and Tables

**Table 1:** Example of process variable groups and sensors specifications.

Groups	Variables	Sensor Type	Unity	Range
Process Cold Water	Pressure	4–20 mA	bar	0 to 10
	Temperature	4–20 mA	${}^{\circ }C$	0 to 100
Clean Dry Air	Pressure	4–20 mA	bar	0 to 10
	Dew Point	4–20 mA	${}^{\circ }C$	–100 to 20
De-ionized Water	Pressure	4–20 mA	bar	0 to 10
	Temperature	4–20 mA	${}^{\circ }C$	0 to 100
	Resistivity	4–20 mA	MOhm	10 to 20
	Flow Rate	4–20 mA	L/min	0 to 50
	pH	4–20 mA	pH	0 to 14
Environment	Temperature	I2C	${}^{\circ }C$	–40 to 80
	Humidity	I2C	%RH	0 to 100
	Particles Concentration	SPI	particles/ft${}^{3}$	5k to 100k class
Nitrogen	Pressure LN2	4–20 mA	bar	0 to 10
	Temperature LN2	4–20 mA	${}^{\circ }C$	–200 to 50
	Pressure N2	4–20 mA	bar	0 to 10
Vaccum	Pressure	4–20 mA	bar	–1 to 0

**Table 2:** Lessons learned and their adaptations.

Area	Detected Problems	Adopted Solution
Administrative	Lack of planning with the customer	Plan and document all requisites to avoid changes in the middle of development
	The lack of ready-made solutions that meet all requirements	Design custom solutions
	Financial limitations to make up a team with super-specialists	Make up a team with few super specialists together with selected students
	The possibility of errors in the development of custom solutions	First make prototypes before the final solutions
Development	Performance difference in hardware components	Simulate and prototype each critical hardware step
	Problems with the operation of some sensors	Buy good sensors and test them in a controlled environment, do not associate all problems with your solution in development
	Different development environments to the RF network	Deploy a small network to identify connection problems early in the factory
	Device operational limitation of wireless MCU	Develop a pilot application to evaluate the limitation due to both network and embedded hardware specs
	Lack of documentation and guides of implementations to embedded hardware	Use devices and operational systems with extensive community support, and examples.
	Data loss in Database development	Perform data backup and replication routines, as well as adopt a versioning solution
	Use of Virtual Machines	Perform tests with possible OS and then install natively
